# 

**Published:** 1827-05

**Authors:** 


### MONTHLY LIST OF MEDICAL BOOKS.

[*No books can be entered on this List except those sent to us for the purpose; as, in the list hitherto transmitted, the names of works have frequently been given as published, which have not appeared for weeks, or even months, after.*]

An Apology for British Anatomy, as an Incitement to the Study of Morbid Anatomy; being an Abstract of a Lecture introductory to a Course of Illustrations of the Morbid Anatomy of the Heart and Aorta, delivered to the Profession in the Autumn of 1826. By RICHARD FARRE, M.D.—London, 1827.

Elements of Physics; or, Natural Philosophy, General and Medical, explained, independently of Technical Mathematics. By N. ARNOTT, M.D. of the Royal College of Physicians.—Pp. 611. London, 1827.

An Essay on Morbid Sensibility of the Stomach and Bowels, as the Proximate Cause, or Characteristic Condition of Indigestion, Nervous Irritability, Mental Despondency, Hypochondriasis, &c. &c. To which are added, Observations on the Diseases and Regimen of Invalids, on their Return from hot and unhealthy Climates. Third Edition. By JAMES JOHNSON, M.D. of the Royal College of Physicians, and Physician to his Royal Highness the Duke of Clarence; Author of the Influence of Tropical Climates on European Constitutions, and Editor of the Medico-Chirurgical Review, &c. &c.—Pp. 157.—London, 1827.

☞ This Essay has come to a third edition in six months. Highly as it has been spoken of by various reviewers, this speaks more highly still.

Observations on the Impropriety of Men being employed in the Business of Midwifery.—Pp. 56. London, 1827.

Laws of Physiology; translated from the Italian of Il Signor Dott. B. MOJON, Professor Emeritus in the Royal University of Genoa, and Member of many learned Bodies. With Additions, and a Physiological Table of Man. (Dedicated by permission to Sir ASTLEY PASTON COOPER, Bart. F.R.S. Surgeon to the King.) By GEORGE R. SKENE, Member of the Royal College of Surgeons in London, and of the Medical and Chirurgical Society, &c. &c. —Pp. 126. London, 1827.

Some Observations on the Medicinal and Dietetic Properties of Green Tea, and particularly on the Controlling Influence it exerts over Irritation of the Brain. By W. NEWNHAM, Esq. Author of an Essay on Inversio Uteri, &c. &c.—Pp. 32. London, 1827.

Cases illustrative of the Beneficial Effects of the Clorurets of Oxides of Sodium and Calcium, of the Chevalier LABARRAQUE, of Paris; being the most powerful and effectual General and Local Antiseptics in the Practice of Physic and Surgery. By J. G. F. HASSELL, M.D. Resident Physician at Bologne-sur-Mer, late of the British Army.—Pp. 31. London, 1827.

### NOTICES.

*It is intended, in some of the following Numbers, to treat of the subjects of which we subjoin a list. We shall feel particularly obliged by the communication of any Cases or Observations connected with these topics.*

Injuries of the Spine.
Diseases of the Heart and Great Vessels.
Wounds, &c. of Arteries.
Removal of Calculi from the Female Bladder.
Hemorrhage from the Urethra.
The application of Moxa to the Treatment of various Chronic Diseases.
Hydrophobia.

---

*Communications have been received from* Dr. LEE, Mr. ROSE, Dr. GREGORY, Mr. WICKHAM, Mr. DICKINSON (through Mr. BRODIE), and Dr. MILLIGAN.

